# The value and role of mosquito meshes in low resource and poor income settings

**DOI:** 10.1007/s10029-020-02323-2

**Published:** 2020-11-01

**Authors:** B. M. Stephenson, A. N. Kingsnorth

**Affiliations:** Hernia International Charity, Plymouth, UK

**Keywords:** Low cost mesh, Inguinal hernia repair

We believe the report by Wiessner et al.[1] is unsound and the data presented does not amount to an indictment as to the use of low cost mesh in low-income countries. The “massive” shrinkage described by the authors after steam sterilization, has not been our experience. We have used AMSA Plastics low-density polyethylene (LDPE) mesh for inguinal hernia repair, in many low-income countries for more than 5 years in over 10,000 patients with results comparable to commercial meshes.

First, the authors did not use any controls for comparison. This is essential to rule out methodological issues during the various pre-sterilization treatments of the meshes. Second, the authors did not describe any measures they took to verify the temperature control of the steam sterilizer. Temperatures inside the device may have exceeded the 121 ^0^C recommended for sterilization of the LDPE mesh. The sterilizer used was apparently of non-medical grade, used commonly for “sterilizing baby bottles”. No self-respecting surgeon would consider implanting a mesh into a patient after “sterilization” by such a ‘Mickey Mouse’ device. Third, the lack of controls means that it is impossible to conclude whether the vigorous pre-sterilization treatment of the meshes with penicillin, streptomycin and amphotericin B had denatured the meshes and rendered them susceptible to the effects of steam and subsequent shrinkage. At this point the reader may be reminded that our own studies found no significant difference when comparing the ultra-structural characteristics and material properties of LDPE mesh to commercial meshes [2].

In rural settings, temperature control of poorly maintained medical-grade steam sterilizers is the most likely cause of occasional mesh shrinkage, as opposed to a change in mesh characteristics. The many Hernia International volunteer surgeons “working in the field” have occasionally describe such events; but these are rare. Nevertheless when observed, the low cost of a piece of LDPE mesh (< US$1.00) means that any shrunken pieces can be discarded to allow safe use with pliable mesh.

Prior to his recent death from COVID-19, the pioneering rural Indian surgeon Ravi Tongaonkar estimated that over 35,000 patients had received affordable hernia repairs with LDPE mesh. We will continue to observe safe clinical practice with this product confident that no patient is exposed to any risk from this material. Furthermore independent reviews and meta-analysis concur as to the safety of low-cost meshes and comparable results with commercial meshes [3, 4].

Finally, whilst the authors suggest that the HerniaSurge guidelines be re-evaluated for patients in low resource and poor income settings, we believe a truly impartial study is needed less hernia surgeons be labeled as complicit in accepting double standards with regards to mosquito mesh hernioplasty. This we all know this is NOT true.
